# Which multi-attribute utility instruments are recommended for use in cost-utility analysis? A review of national health technology assessment (HTA) guidelines

**DOI:** 10.1007/s10198-020-01195-8

**Published:** 2020-06-08

**Authors:** Matthew Kennedy-Martin, Bernhard Slaap, Michael Herdman, Mandy van Reenen, Tessa Kennedy-Martin, Wolfgang Greiner, Jan Busschbach, Kristina S. Boye

**Affiliations:** 1 Kennedy Martin Health Outcomes Ltd, Suite 404, The Dock Hub, Wilbury Villas, Hove, BN3 6AH UK; 2 Department of Psychiatry, Section of Medical Psychology and Psychotherapy, Erasmus MC, Rotterdam, The Netherlands; 3 EuroQol Research Foundation, Rotterdam, The Netherlands; 4 Office of Health Economics (OHE), London, UK; 5 Department of Health Economics at the School of Public Health, Bielefeld University, Bielefeld, Germany; 6 Eli Lilly and Company, Indianapolis, IN USA

**Keywords:** Health technology assessment, Cost-utility analysis, Multi-attribute utility instruments, Pharmacoeconomics, Guidelines, Utility, i11, i18

## Abstract

**Background:**

Several multi-attribute utility instruments (MAUIs) are available from which utilities can be derived for use in cost-utility analysis (CUA). This study provides a review of recommendations from national health technology assessment (HTA) agencies regarding the choice of MAUIs.

**Methods:**

A list was compiled of HTA agencies that provide or refer to published official pharmacoeconomic (PE) guidelines for pricing, reimbursement or market access. The guidelines were reviewed for recommendations on the indirect calculation of utilities and categorized as: a preference for a specific MAUI; providing no MAUI preference, but providing examples of suitable MAUIs and/or recommending the use of national value sets; and recommending CUA, but not providing examples of MAUIs.

**Results:**

Thirty-four PE guidelines were included for review. MAUIs named for use in CUA: EQ-5D (*n* = 29 guidelines), the SF-6D (*n* = 11), HUI (*n* = 10), QWB (*n* = 3), AQoL (*n* = 2), CHU9D (*n* = 1). EQ-5D was a preferred MAUI in 15 guidelines. Alongside the EQ-5D, the HUI was a preferred MAUI in one guideline, with DALY disability weights mentioned in another. Fourteen guidelines expressed no preference for a specific MAUI, but provided examples: EQ-5D (*n* = 14), SF-6D (*n* = 11), HUI (*n* = 9), QWB (*n* = 3), AQoL (*n* = 2), CHU9D (*n* = 1). Of those that did not specify a particular MAUI, 12 preferred calculating utilities using national preference weights.

**Conclusions:**

The EQ-5D, HUI, and SF-6D were the three MAUIs most frequently mentioned in guidelines. The most commonly cited MAUI (in 85% of PE guidelines) was EQ-5D, either as a preferred MAUI or as an example of a suitable MAUI for use in CUA in HTA.

## Introduction

Several methods of economic evaluation are utilized in health technology assessment (HTA), including cost-utility analysis (CUA), a form of cost-effectiveness analysis that assesses the value of interventions, typically according to the incremental cost per quality-adjusted life-year (QALY). A patient’s health state preferences may be measured directly to derive utilities, using methods such as standard gamble (SG) or time trade-off (TTO). Utilities may also be determined indirectly by means of generic or disease-specific preference-based questionnaires, with responses mapped onto a utility scale using an algorithm that attaches weights—generally derived from societal preferences for health states. Generic multi-attribute utility instruments (MAUIs) are commonly used for the indirect measurement of utilities. Several MAUIs are available for indirect measurement of utilities in CUA, including the EQ-5D (two versions: EQ-5D-3L and EQ-5D-5L) [1], the Short-Form 6-Dimension (SF-6D) [2], the Health Utilities Index (two versions: HUI2 and HUI3) [3], Assessment of Quality of Life (several versions, e.g. AQoL 6D and 8D) [4], 15D [5], VR-6D [6] and the Quality of Well-Being (QWB) instrument [7]. Each MAUI has its own descriptive health classification system and preference-based algorithm used to derive utility scores [8].

Official pharmacoeconomic (PE) guidelines inform manufacturers and others on which methods to follow with respect to CUA to support applications for access, reimbursement, or pricing. Understanding these recommended methods is important to facilitate planning for studies and gain a better appreciation of the needs of decision-makers. There is no international consensus about the content of PE guidelines, so recommendations differ among countries around the world [9].

As such, the objective of this review was to identify recommendations from official national PE guidelines about the use of MAUIs within CUA; in addition, the review sought to understand in which countries national preference weights (value sets) were required for the determination of utilities using a MAUI.

## Methods

### HTA agency and PE guideline search

The initial step in the review process involved the identification of national HTA agencies worldwide. The following databases were reviewed: the National Institute for Health Research (NIHR) HTA database; the International Society for Pharmacoeconomics and Outcomes Research (ISPOR) PE guidelines; the International Network of Agencies for Health Technology Assessment (INAHTA); the World Health Organization; the European Network for HTA (EuNetHTA); HTAsiaLink; and Health Technology Assessment International.

Once HTA agencies had been identified, their webpages were reviewed to determine whether they utilized publicly available PE guidelines (or outlined PE guidelines within their submission guidance documents). This assessment was further informed by searches on PubMed and Google, as well as the ISPOR PE guidelines database. Where these searches suggested relevant official PE guidelines were available, but these remained elusive, help was sought to obtain them from local health economic experts.

### Identification of CUA MAUI requirement or recommendation

PE guidelines were included for countries where HTA is used to inform the decision-making process for pricing, reimbursement or market access for medicines by the national healthcare decision-making body. This definition is similar to the one used by ISPOR in their PE guidelines database (https://tools.ispor.org/peguidelines/). Multinational guidelines (e.g. Mercosur) and subnational guidelines (e.g. Catalonia) were excluded.

Once the latest versions of these guidelines were identified, they were reviewed to determine whether they recommended the use of CUA as a method for economic evaluation. If CUA is recommended, the PE guidelines were then reviewed to determine whether specific MAUIs were preferred; and if none were preferred, whether examples of MAUIs were provided and whether the use of national preference weights (value sets) were recommended. When clarification was required regarding the status or content of PE guidelines or help was needed with the translation of relevant guideline sections, input was sought from local health economic experts. The focus of this review was only on indirect methods for deriving utilities within CUA. This method generally involves applying utility algorithms to generic or disease-specific preference-based questionnaires; guidelines relating to non-MAUI methods such as mapping were excluded. The focus of the review was on pharmaceutical guidelines. Any guidelines relating specifically to medical devices or technology were considered outside scope. The searches to inform this review were undertaken between January and March 2019, with additional research and expert input gathered until August 2019.

## Results

### Guideline selection

**Fig. 1:**
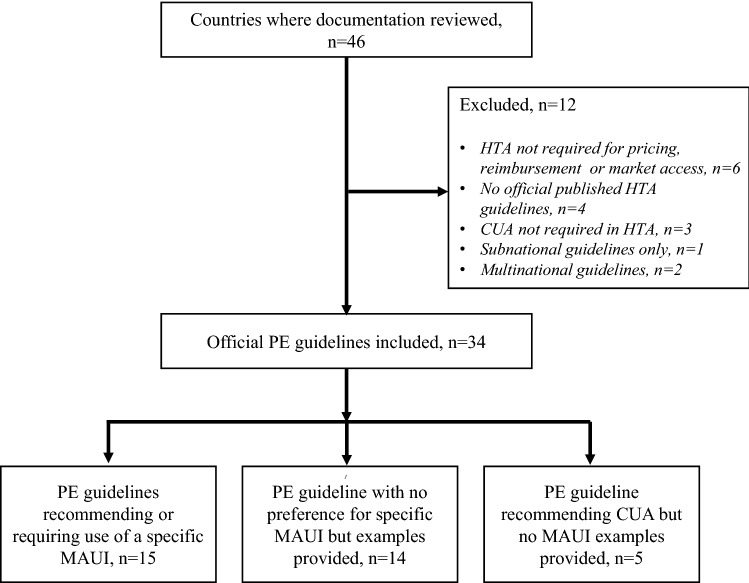
Flow chart for inclusion of PE guidelines in the review. *CUA* cost-utility analysis, *HTA* health technology assessment, *MAUI* multi-attribute utility instrument, *PE* pharmacoeconomic

Documentation from 46 countries was reviewed and 12 were excluded in line with eligibility criteria, as presented in Fig. 1 (Argentina, Austria, Baltic States, Germany, Italy, Kazakhstan, Romania, Spain, Switzerland, Tunisia, United States, and Uruguay; reasons for the exclusion provided in Table 1), leaving 34 official guidelines, which are summarized in this report. The 34 included guidelines were categorized as those that preferred or encouraged the use of a specified MAUI (Table 2) and guidelines that recommended CUA but recorded no preference for a specific MAUI (Table 3).

**Table 1:** Countries excluded from the review

	Reason for exclusion				
	National PE HTA not required for pricing, reimbursement or market access decision-making	No “official” published national PE HTA guidelines	CUA not required in PE HTA	Subnational guidelines only	Use multinational guidelines
Argentina	$\checkmark$				
Austria	$\checkmark$				
Baltic States					$\checkmark^{\text a}$
Germany			$\checkmark$		
Italy	$\checkmark$				
Kazakhstan		$\checkmark$			
Romania			$\checkmark$		
Spain		$\checkmark$		$\checkmark$	
Switzerland	$\checkmark$		$\checkmark$		
Tunisia	$\checkmark$	$\checkmark$			
United States	$\checkmark$				
Uruguay		$\checkmark$			$\checkmark^{\text b}$

*CUA* cost-utility analysis, *HTA* health technology assessment, *PE* pharmacoeconomic

^a^2002 Baltic Guideline for Economic Evaluation of Pharmaceuticals

^b^2015 MERCOSUR (The Southern Common Market) guidelines usually followed

**Table 2:** Official PE guidelines that prefer or encourage the use of a specific MAUI for CUA, either alone or with another MAUI

Country	Agency	Guideline	Year	Recommended or preferred MAUI	Reason for preference	Notes/observations of relevance
*Prefer/encourage use of a single MAUI*						
Belgium	Belgian Health Care Knowledge Centre	Belgian Guidelines for Economic Evaluations and Budget Impact Analyses, 2nd Edition	2012	EQ-5D	“In order to stimulate the use of generic utility instruments and to promote consistency”	Use of Belgian preference values is preferred
Bulgaria	National Center for Public Health and Analysis	Health Technology Assessment Guidelines	2018	EQ-5D-3L EQ-5D-5L	“…it [EQ-5D] is commonly used, it allows the greatest comparability of the results of economic analyses.”	
Columbia	El Instituto de Evaluación Tecnológica en Salud (IETS) (Institute of Health Technology Assessment)	Manual Para la Elaboración de Evaluaciones Económicas en Salud (Manual for the Preparation of Economic Evaluations in Health)	2014	EQ-5D-3L	Not stated	Preferences from Latino population in USA should be used
Croatia	Agency for Quality and Accreditation in Health Care	Croatian Guideline for Health Technology Assessment Process and Reporting	2011	EQ-5D	Not stated	National preferences required
Czech Republic	Státní Ústav pro Kontrolu Léčiv (State Institute for Drug Control)	Cost-Effectiveness Analysis Critical Appraisal Procedure	2017	EQ-5D	“A pharmacoeconomic evaluation always has to apply the same method of measuring quality of life to all (clinical) conditions, as individual methods are not mutually comparable and result in varying partial values of utility.”	Preference to use Czech health preferences, but if not available, use utilities from the UK
England	National Institute for Health and Care Excellence (NICE)	Guide to the Methods of Technology Appraisal (PMG9) NICE. Position Statement on Use of the EQ-5D-5L Valuation Set for England	2013 2018	EQ-5D-3L EQ-5D-5L	“Different methods used to measure health-related quality of life produce different utility values; therefore, results from different methods or instruments cannot always be compared. Given the need for consistency across appraisals, one measurement method, the EQ-5D, is preferred for the measurement of health-related quality of life in adults.”	Use UK 3L-value set with mapping if 5L descriptive system used
The Netherlands	Zorginstituut Nederland	Dutch National Guideline for Economic Evaluations in Healthcare	2016	EQ-5D-5L	“In view of the possible differences in the assessment of quality of life that may arise from the use of different questionnaires, quality of life should consistently be measured with the EQ-5D-5L and should be valued using Dutch reference values.”	Use Dutch preference values
New Zealand	Pharmaceutical Management Agency (PHARMAC)	Prescription for Pharmacoeconomic Analysis. Methods for Cost-Utility Analysis (Version 2.2)	2015	EQ-5D	“The EQ-5D is widely used internationally and utility weights have been derived from the New Zealand population. Therefore, PHARMAC recommends referring to the EQ-5D Tariff 2 first and using it to describe the health states.”	Use New Zealand Tariff 2
Norway	Norwegian Medicines Agency	Guidelines for the submission of documentation for single technology assessment of pharmaceuticals	2018	EQ-5D-3L EQ-5D-5L	“To make comparison between different single technology assessments possible, EQ-5D must, as a rule, be used.”	For consistency, the results from 3 and 5L should be converted to a comparable set of values (mapped to 3L values). The UK population-based tariff is recommended until a more relevant and applicable (Norwegian) tariff is available
Poland	Agencja Oceny Technologii Medycznych (AOTMiT) (Agency for Health Technology Assessment and Tariff System)	Health Technology Assessment Guidelines (Version 3.0)	2016	EQ-5D-3L EQ-5D-5L	“…since it is commonly used, it allows for the greatest comparability of the results of economic analyses.”	Use Polish 3L value set and crosswalk until 5L value set is available
Portugal	Ministério da Saúde	Portaria (Ordinance) no. 391/2019. Sumário: Aprova os princípios e a caraterização das Orientações Metodológicas para Estudos de Avaliação Económica de Tecnologias de Saúde	2019	EQ-5D-5L	Not stated	Use Portuguese tariffs
Scotland	Scottish Medicines Consortium (SMC)	Guidance to Submitting Companies for Completion of New Product Assessment Form	2019	EQ-5D	“Given the comparative nature of the SMC’s work and the need for consistency across appraisals, the SMC would ideally wish that all appraisals used the same system.”	Guideline notes, EQ-5D appears to be the most appropriate choice in the UK
Thailand	Health Intervention and Technology Assessment Program (HITAP), Ministry of Public Health	Guidelines for Health Technology Assessment in Thailand (Second Edition)	2014	EQ-5D-3L^a^	“…due to the validity, reliability, responsiveness, feasibility and availability of the established value set for Thai population.”	Use Thai value set
*Preference for more than one MAUI*						
Chile	Ministerio de Salud de Chile	Guía Metodológica para la Evaluación Económica de Intervenciones en Salud en Chile (Methodological Guide for the Economic Evaluation of Health Interventions in Chile)	2013	EQ-5D DALY	There is a Chilean social valuation of EQ-5D health states National researchers are familiar with DALYs following burden of disease studies in Chile	Chilean preferences used
France	Haute Autorité de Santé (French National Authority for Health)	Choices in Methods for Economic Evaluation: A Methodological Guide	2012	EQ-5D HUI	“…in order to promote the consistency and comparability across CUAs. They are the only ones, to date, with a set of preferences values obtained from a representative sample of the French population.”	Use validated French preference values

*CUA* cost-utility analysis, *DALY* disability-adjusted life-year, *HUI* Health Utilities Index, *MAUI* multi-attribute utility instrument, *SF-6D* Short-Form 6-Dimension

^a^The Thailand guideline states that at the time of publication in 2014, no EQ-5D-5L value set from the Thai population was available (although it noted that HITAP was working on one with the EuroQol Group); consequently, the EQ-5D-3L is the preferred method used to measure utility. Note, in 2018, the EQ-5D-5L value set for Thailand was published [10]

**Table 3:** Official PE guidelines that recommend CUA as an economic evaluation approach, including the use of QALYs, but provide no preference for a specific MAUI

Country/region	Agency	Guideline	Year	MAUIs named as examples	Utility weights based on preferences of domestic population preferred
Australia	Pharmaceutical Benefits Advisory Committee, Department of Health and Ageing, Australian Government (PBAC)	Guidelines for Preparing a Submission to the Pharmaceutical Benefits Advisory Committee (Version 5.0)	2016	EQ-5D (3L and 5L) HUI2 or HUI3 SF-6D AQoL CHU9D	$\checkmark$
Brazil	Ministério da Saúde	Diretrizes Metodológicas: Diretriz de Avaliação Econômica: 2nd Edition (Methodological Guideline: Economic Assessment Guideline)	2014	EQ5D SF-6D HUI QWB	$\checkmark$
Canada	Canadian Agency for Drugs and Technologies in Health (CADTH)	Guidelines for the Economic Evaluation of Health Technologies: Canada (4th Edition)	2017	EQ-5D HUI SF-6D	$\checkmark$
Cuba	Ministry of Health	Methodological Guidelines for Health Economic Evaluation in Healthcare	2003	None^a^	Not stated
Egypt	Ministry of Health; Egyptian Drug Authority	Guidelines for Reporting Pharmacoeconomic Evaluations	2013^b^	EQ-5D SF-6D	Unclear^c^
Finland	Lääkkeiden hintalautakunta (Hila; Pharmaceuticals Pricing Board, Ministry of Social Affairs and Health)	Submission guideline: Preparing a health economic evaluation to be attached to the application for reimbursement status and wholesale price for a medicinal product	2018	None	Not stated
Hungary	National institute for Pharmacy and Nutrition (OGYÉI)	Professional healthcare guideline on the methodology of health technology assessment	2017	EQ-5D SF-6D	$\checkmark$
Iran^d^	Iran Ministry of Health and Medical Education Iran Food and Drug Administration National committee for selecting and registering medicine	Regulation of Submitting Applications for Medicine with Regard to Development of Economic Evaluation	2016	EQ-5D SF-6D SF-36	$\checkmark$
Ireland	Health Information and Quality Authority	Guidelines for the Economic Evaluation of Health Technologies in Ireland	2018	EQ-5D (3L and 5L) SF-6D	$\checkmark$
Israel	Ministry of Health – Pharmaceutical Administration	Guidelines for the submission of a request to include a pharmaceutical product in the national list of health services	2010	None	From a similar population^e^
Japan	Center for Outcomes Research and Economic Evaluation for Health, National Institute of Public Health (C2H)	Guideline for Preparing Cost-Effectiveness Evaluation to the Central Social Insurance Medical Council	2019	EQ-5D SF-6D HUI	$\checkmark$
Malaysia	Ministry of Health	Pharmacoeconomic Guideline for Malaysia	2012	EQ-5D HUI3 SF-6D	$\checkmark$
Mexico^f^	Consejo de Salubridad General	Guide for Conducting Economic Evaluation Studies for Updating the National Formulary in Mexico	2017	None	$\checkmark$
Singapore	Agency for Care Effectiveness	Drug Evaluation Methods and Process Guide	2018	EQ-5D-5L SF-6D HUI3 AQoL	$\checkmark$
Slovak republic	Ministry of Health	Metodická pomôcka pre vykonávanie farmako-ekonomického rozboru lieku, medicínsko-ekonomického rozboru zdravotníckej pomôcky a medicínsko-ekonomického rozboru dietetickej potraviny Decree no. 422/2011: Vyhláška Ministerstva zdravotníctva Slovenskej republiky o podrobnostiach farmako-ekonomického rozboru lieku	2012	EQ-5D HUI QWB	Not stated
Slovenia	Health Insurance Institute of Slovenia	Rules of Reimbursement. Official Gazette of the Republic of Slovenia, No 35/2013	2013	None	Not stated
South Korea^g^	Health Insurance Review and Assessment Service (HIRA)	Guidelines for Economic Evaluation for Pharmaceuticals: Second Version	2011	EQ-5D^h^ HUI QWB	$\checkmark$
Sweden	Dental and Pharmaceutical Benefits Agency (TLV)	Ändring i Tandvårds-och Läkemedelsförmånsverkets Allmänna Råd (TLVAR 2003:2) om Ekonomiska Utvärderinga (Change in the Dental and Drug Benefits Agency’s General Advice [TLVAR 2003: 2] on Financial Evaluations)	2017	EQ-5D	✖^i^
Taiwan^j^	Center for drug evaluation	Methodological guidelines for health technology assessment	2014	EQ-5D SF-6D HUI	$\checkmark$

*AQoL* Assessment of Quality of Life; *CHU9D*, Child Health Utility 9D index; *HUI*, Health Utilities Index; *MAUI*, multi-attribute utility instrument; *PE,* pharmacoeconomic; *QWB*, Quality of Well-Being; *SF-36*, Short-Form 36-Item Health Survey; *SF-6D*, Short-Form 6-Dimension

^a^ QALYs are defined in the guidelines under the heading of useful concepts for the application of health economic evaluations, but their use is not discussed further

^b^ Date uncertain; data from ISPOR website rather than the original source

^c^ States that valuation of these changes in the health state should then be reported for the general population

^d^ HTA is required when an application is submitted to the Iranian FDA for a new medicine to be included in the Iranian Medicines List; this does not necessarily mean, however, that it will be added to the Iran Health Benefit Package or be covered by insurers, only that it is available for prescription

^e^ Guideline states that it is preferable to use values from populations similar to that of Israel

^f^ Economic evaluation is mandatory for health technologies submission to National Formulary, but CUA is a complementary, not mandatory, component

^g^ HTA is not mandatory and is only required when a manufacturer hopes to negotiate a higher price (Personal communication, email from Prof. N. Luo, 17 Apr 2019)

^h^ Information source is article by authors from HIRA that summarizes content of 2011 Korean guidelines [11]. MAUI examples were provided in the article

^i^ Preference for weightings based on persons in the health condition in question

^j^ HTA is not mandatory for all drugs. It is required when a drug is anticipated to have a large budget impact or when a manufacturer hopes to negotiate a higher price (Personal communication, email from Prof. N. Luo, 17 Apr 2019)

### MAUI instruments mentioned in official PE guidelines

**Fig. 2:**
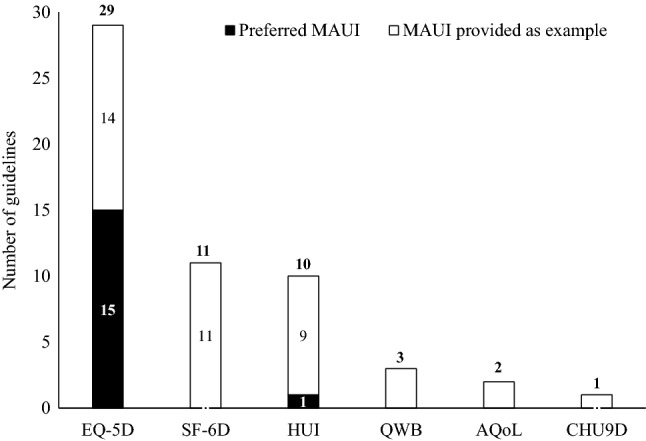
MAUIs preferred or provided as an example across identified official PE guidelines. *AQoL* Assessment of Quality of Life, *CHU9D* Child Health Utility 9D, *HUI* Health Utility Index, *MAUI* multi-attribute utility instrument, *QWB* quality of well-being, *SF-6D* Short-Form 6-Dimension. Numbers sum to more than 34 because some guidelines cite more than one MAUI

In the 34 guidelines included in the review, the following MAUIs were named for use in CUA: EQ-5D (cited in *n* = 29 guidelines), the SF-6D (*n* = 11 guidelines), HUI (*n* = 10), QWB (*n* = 3), AQoL (*n* = 2), and Child Health Utility 9D index (CHU9D) (*n* = 1) (Fig. 2). Although not MAUIs, for completeness, it should be noted that both the Short-Form 36-Item Health Survey (SF-36) (*n* = 1) and use of the disability-adjusted life-year (DALY) (*n* = 1) were also grouped with MAUIs in two of the guidelines (from Iran and Chile, respectively).

### Official PE guidelines preferring/encouraging use of a specific MAUI

A number of official PE guidelines (*n* = 15) recommended the use of a specific MAUI; these are listed in Table 2. Only one instrument, the EQ-5D, was included as a preferred MAUI in all 15 guidelines. It was the only preferred MAUI in 13 of these guidelines, and in a further two it was preferred along with a second instrument (Table 2; Fig. 2). Most of these guidelines did not provide a preference for which EQ-5D version to use. Six recommended using the EQ-5D-3L. The EQ-5D-5L, which has only been available for a few years, is recommended as an alternative to the EQ-5D-3L in four and preferred in two guidelines (The Netherlands and Portugal) (Table 2). In one of the two guidelines where EQ-5D or another instrument were cited as preferred, the other utility instrument was the HUI (*n* = 1, France) (Table 2). In the Chilean guidelines, DALYs were cited as being an alternative to the EQ-5D; however, disability weights are different from utilities and not derived using a MAUI. This observation is included in the results (Table 2) for completeness. None of the identified guidelines preferred a MAUI other than the EQ-5D, without also recommending the EQ-5D.

### Official PE guidelines with no preference for a specific MAUI but including named examples

Of the official PE guidelines identified in the search, 14 expressed no preference for a specific MAUI but did provide examples of acceptable instruments within their recommendations (Table 3). A range of examples was provided, with the EQ-5D being the most frequently cited MAUI (cited in all 14 guidelines, three of which cited the new EQ-5D-5L). The next most common MAUI examples were the SF-6D and HUI in 11 and nine guidelines, respectively; the QWB in three guidelines, the AQoL in two guidelines; and the CHU9D in one guideline (Table 3). In the Iranian guidelines, there is a reference to the SF-36, but this is not a MAUI and our assumption is that they will accept a mapping from the SF-36 to the SF-6D.

### Official PE guidelines that recommend the use of national preference weights to calculate utilities

Of the guidelines that did not state a preference for a specific MAUI (Table 3), most (*n* = 12) recommended that calculation of utility weights should be based on preferences from the domestic population.

### Official PE guidelines that recommend CUA but do not provide MAUI examples

Five guidelines were identified that did recommend economic evaluation by CUA but did not provide any examples of acceptable MAUIs (Table 3). These included guidelines issued in Cuba, Finland, Israel, Mexico, and Slovenia.

## Discussion

The objective of this review was to provide an overview of recommendations from HTA agencies on the use of MAUIs in CUA. As far as we are aware, this is the first published review to comprehensively summarize the contents of HTA guidelines relating to the use of MAUIs in CUA around the world. Previous reviews, such as the 2017 study by Rowen and colleagues [9], have also explored this topic but restricted themselves to specific countries/regions (Australia, Canada, Catalonia, England and Wales, France, Germany, The Netherlands, Scotland, Sweden). In a 2016 review of the use of EQ-5D in Central and Eastern Europe, Rencz and colleagues [12] noted the countries where EQ-5D is recommended in HTA guidelines. Others have taken a much broader approach in their summaries of HTA guidelines and only briefly consider recommendations on MAUIs [13].

Six MAUIs were recommended or cited in guidelines; EQ-5D, HUI, and SF-6D were the most frequently mentioned, with EQ-5D found to be the most dominant measure. Of the 34 sets of guidelines from around the world that were identified in the review, EQ-5D was mentioned in 85% (*n* = 29) as a preferred instrument for the determination of health utilities or as an example of a suitable instrument. Whenever a guideline-recommended specific MAUIs (*n* = 15 guidelines), EQ-5D was found to be the only preferred instrument in 13 guidelines and one of two preferred instruments, along with another MAUI or the DALY, in the remaining two guidelines. No other MAUI came close to this level of prominence. Reasons provided in some of the PE guidelines for preferring a particular MAUI include that EQ-5D is a commonly used instrument enabling consistency and comparability between data sets, and that a national value set is available (Table 2).

The dominance of EQ-5D as a MAUI used in clinical studies mirrors the preferences provided in the PE guidelines. A review of articles listed on the Web of Science between 2005 and 2010 identified 1663 studies that had included a MAUI [14]. Of these, 63% used EQ-5D; 15% the HUI2 or HUI3; 9% the SF-6D; and the remaining 15% used the 15D, QWB, or AQoL.

Most of the guidelines referred to EQ-5D in general or to the EQ-5D-3L version. Some of these PE guidelines are several years old, and therefore the number citing the EQ-5D-5L—developed to increase sensitivity (discriminatory power) while maintaining ease of use [15]—remains relatively low.

Of the 19 PE guidelines that recommend CUA, but provide no preference for a specific MAUI, 12 included a preference for MAUIs utilizing national value sets. The use of some MAUIs could, therefore, be limited in PE analysis in these countries, since preference weights in the national general population may not be available.

It is interesting to note that official PE guidelines were available from only 34 countries that specified the use of QALYs for use in CUA within economic evaluations. Although this may in part reflect policy decisions by a few governments to use different methods to assess the value of medications (e.g. in Germany), in other countries the lack of detailed published guidelines is more likely to reflect the current more nascent state of their HTA systems. However, as resources available for public healthcare continue to be stretched around the globe, it will be increasingly important for policymakers to be supported with the best available evidence on new and existing medications to make informed choices with respect to resource allocation [16]. Consequently, the HTA environment will continue to develop, most notably as countries that did not previously have systems in place (e.g. in parts of Eastern Europe, Latin America, and Asia) begin to develop and implement them [17–20]. As these HTA systems evolve, more official PE guidelines will be developed and the number recommending the use of indirect methods for deriving utilities within CUA can be expected to grow accordingly.

Guidelines relating specifically to medical devices or technology were considered outside the scope of this review. However, as in the present study, a recently published review of European HTA guidelines for medical devices also found that EQ-5D was the most frequently mentioned MAUI and it was the preferred measure in most national HTA guidelines [21].

While the current review provides some interesting insights into recommendations on MAUI use in official PE guidelines, the findings must be interpreted within the limitations of the study. Although a wide range of sources was reviewed, and references cross-checked, some guidelines may have been overlooked. Likewise, some of the guidelines were not available in English, Dutch, or German, necessitating online translation. Although such translations were validated by local experts, there is always a risk that some ambiguity remains. In some cases, it was also unclear which guideline from a particular country should be used and included in the review; and it may be that even in countries where official PE guidelines do exist, these are not published or publicly available and would not have been identified by the current searches. A further limitation of the review is that supplemental informal guidance may be provided by HTA authorities in addition to that published in official guidelines.

Finally, it is important to recognize that the HTA environment is continually evolving and an overview of the sort provided here can quickly become outdated. For example, several countries (e.g. Argentina) are considering developing new HTA structures, and existing PE guidelines will also be refined as the science of economic evaluation evolves. Regular updating of the review is, therefore, required.

## Conclusions

Published official PE guidelines from around the world were identified in the current review. There appears to be substantial consensus among them in terms of choice of MAUI instruments, and three instruments (EQ-5D, HUI, SF-6D) are each cited in at least 10 country guidelines. By far the most common was the EQ-5D, which was cited in 85% of PE guidelines either as the preferred MAUI or as an example of a suitable MAUI for use in CUA in HTA economic evaluations. The preference for EQ-5D in guidelines was variously described as being due to its widespread use in studies, enabling consistency and comparability, and the availability of national value sets. Where PE guidelines provided examples of MAUIs but did not give a preference, a majority explicitly recommended the use of national value sets for the determination of utilities.

This review provides an overview of the global picture on preferences for the use of the MAUIs in official PE guidelines. It also provides insight for stakeholders seeking to understand what instruments are used in HTA across different countries, and for those developing HTA systems and PE guidelines in countries that have not previously been part of the landscape.

## Appendix

### National guidelines included in the survey

Agencja Oceny Technologii Medycznych (Poland): Health technology assessment guidelines (version 3.0). https://www.aotm.gov.pl/www/wp-content/uploads/wytyczne_hta/2016/20161104_HTA_Guidelines_AOTMiT.pdf (2016) Accessed 11 June 2019

Agency for Care Effectiveness (Singapore): Drug evaluation methods and process guide. https://www.ace-hta.gov.sg/public-data/our-process-and-methods/ACE%20methods%20and%20process%20guide%20for%20drug%20evaluation%20(5%20Feb%202018).pdf (2018) Accessed 11 June 2019

Agency for Quality and Accreditation in Health Care (Croatia): Croatian guideline for health technology assessment process and reporting. https://aaz.hr/sites/default/files/hrvatske_smjernice_za_procjenu_zdravstvenih_tehnologija.pdf (2011) Accessed 11 June 2019

Assembly of the Health Insurance Institute (Slovenia): Rules on the classification of medicine on the list. https://tools.ispor.org/PEguidelines/countrydet.asp?c=42&t=1 (2013) Accessed 11 June 2019.

Belgian Health Care Knowledge Centre: Belgian guidelines for economic evaluations and budget impact analyses (2nd ed). https://kce.fgov.be/sites/default/files/atoms/files/KCE_183_economic_evaluations_second_edition_Report_update.pdf (2012) Accessed 11 June 2019

Canadian Agency for Drugs and Technologies in Health: Guidelines for the economic evaluation of health technologies: Canada (4th ed). https://cadth.ca/dv/guidelines-economic-evaluation-health-technologies-canada-4th-edition (2017) Accessed 11 June 2019

Center for Drug Evaluation (Taiwan): Methodological guidelines for health technology assessment. https://tools.ispor.org/PEguidelines/source/HTA_guidelines_Taiwan.pdf (2014) Accessed 11 June 2019

Center for Outcomes Research and Economic Evaluation for Health, National Institute of Public Health (C2H) (Japan): Guideline for preparing cost-effectiveness evaluation to the Central Social Insurance Medical Council (Version 2.0). https://c2h.niph.go.jp/tools/guideline/guideline_en.pdf (2019) Accessed 21 July 2019.

Consejo de Salubridad General (Mexico): Guia para la conducción de estudios de evaluación económica para la actualización del cuadro básico y catálogo de insumos del sector salud en México [Guide for conducting economic evaluation studies for updating the national formulary in Mexico]. https://www.csg.gob.mx/contenidos/priorizacion/cuadro-basico/guias/guias.html (2017). Accessed 11 June 2019.

Dental and Pharmaceutical Benefits Agency (TLV) (Sweden): [Change in the Dental and Drug Benefits Agency's general advice [TLVAR 2003: 2] on financial evaluations]. https://www.tlv.se/download/18.467926b615d084471ac3230c/1510316374332/TLVAR_2017_1.pdf (2017) Accessed 11 June 2019.

El Instituto de Evaluación Tecnológica en Salud (Colombia): [Manual for the preparation of economic evaluations in health]. https://www.iets.org.co/Manuales/Manuales/Manual%20evaluación%20económica%20web%2030%20sep.pdf (2014). Accessed 11 June 2019.

Haute Autorité de Santé (France): Choices in methods for economic evaluation: a methodological guide. https://www.has-sante.fr/portail/upload/docs/application/pdf/2012-10/choices_in_methods_for_economic_evaluation.pdf (2012) Accessed 11 June 2019.

Health Information and Quality Authority (Ireland): Guidelines for the economic evaluation of health technologies in Ireland. https://www.hiqa.ie/sites/default/files/2018-01/HIQA_Economic_Guidelines_2018.pdf (2018) Accessed 11 June 2019.

Health Insurance Review and Assessment Service (South Korea): Guidelines for economic evaluation for pharmaceuticals: 2nd version (2011) Reviewed In: Bae S et al.: Korean guidelines for pharmacoeconomic evaluation (second and updated version): consensus and compromise. Pharmacoeconomics. 31(4), 257–267 (2013).

Health Intervention and Technology Assessment Program (Thailand): Ministry of Public Health guidelines for health technology assessment in Thailand (2nd ed).

Iran Ministry of Health; Iran Food and Drug Administration; National Committee for Selecting and Registering Medicine: Regulation of submitting applications for medicine with regard to development of economic evaluation. https://fda.gov.ir/uploads/073a4a691823574a6a271953b1e2b100.pdf (2016) Accessed 11 June 2019.

Lääkkeiden Hintalautakunta (Finland): Preparing a health economic evaluation to be attached to the application for reimbursement status and wholesale price for a medicinal product. https://www.hila.fi/c/document_library/get_file?folderId=793451&name=DLFE-10632.pdf (2018) Accessed 11 June 2019.

Ministério da Saúde (Portugal).[Portaria (Ordinance) no. 391/2019. Sumário: Aprova os princípios e a caraterização das Orientações Metodológicas para Estudos de Avaliação Económica de Tecnologias de Saúde]. https://dre.pt/web/guest/pesquisa/-/search/125815921/details/normal?l=1 (2019) Accessed: 17 Dec 2019.

Ministerio de Salud de Chile: [Methodological guide for the economic evaluation of health interventions in Chile]. https://www.orasconhu.org/case/sites/default/files/files/EE_FINAL_web.pdf (2013). Accessed 4 June 2019.

Ministry of Health (Brazil): [Methodological guideline: economic assessment guideline: 2nd ed]. https://bvsms.saude.gov.br/bvs/publicacoes/diretrizes_metodologicas_diretriz_avaliacao_economica.pdf (2014) Accessed 11 June 2019.

Ministry of Health (Cuba): Guía metodológica para la evaluación económica en salud. https://tools.ispor.org/PEguidelines/source/Methodological-Guidelines-for-Health-Economic-Evaluations-in-Cuba.pdf (2003) Accessed 9 August 2019.

Ministry of Health; Egyptian Drug Authority: Guidelines for reporting pharmacoeconomic evaluations. https://www.eda.mohealth.gov.eg/Files/402_Egyptian_Pharmacoeconomic_guidelines.pdf (2013) Accessed 11 June 2019.

Ministry of Health (Malaysia): Pharmacoeconomic guideline for Malaysia. https://www.pharmacy.gov.my/v2/sites/default/files/document-upload/pharmacoeconomic-guideline-malaysia.pdf (2012) Accessed 11 June 2019.

Ministry of Health – Pharmaceutical Administration (State of Israel): Guidelines for the submission of a request to include a pharmaceutical product in the national list of health services. https://tools.ispor.org/PEguidelines/source/Israel-Guidelines-for-submission_2010.pdf (2010) Accessed 9 August 2019.

Ministry of Health of the Slovak Republic: Decree regarding pharmaco-economic analysis of drugs (Vyhláška Ministerstva zdravotníctva Slovenskej republiky o podrobnostiach farmako-ekonomického rozboru lieku č. 422/2011 Z. z.). Slovak Republic. https://www.zakonypreludi.sk/zz/2011-422 (2011) Accessed 9 August 2019.

Ministry of Health of the Slovak Republic: Methodological tool for the implementation of economic analysis of pharmaceuticals and medical devices and dietetic food. (in Slovak: Metodická pomôcka pre vykonávanie farmako-ekonomického rozboru lieku, medicínsko-ekonomického rozboru zdravotníckej pomôcky a medicínsko-ekonomického rozboru dietetickej potraviny). Slovak Republic. https://www.health.gov.sk/?Dokumenty-Farmako-ekonomicky-a-medicinsko-ekonomicky-rozbor (2012) Accessed 9 August 2019.

Ministry of Human Resources (Hungary): Az Emberi Eroforrások Minisztériuma szakmai irányelve az egészségügyi technológia értékelés módszertanáról és ennek keretében költséghatékonysági elemzések készítésérol (The Technical Guideline on the Methodology of Health-Economic Analyses and Conducting Cost-Effectiveness Analyses by the Ministry of Human Resources), Egészségügyi Közlöny 2017/3. 821–842. https://www.hbcs.hu/uploads/jogszabaly/2481/fajlok/egeszsegugyi_technologia_ertekeles.pdf (2017) Accessed 9 August 2019.

National Center for Public Health and Analysis (Bulgaria): Health technology assessment guidelines. (2018).

National Institute for Health and Care Excellence: Guide to the methods of technology appraisal (PMG9). https://www.nice.org.uk/process/pmg9/chapter/foreword (2013) Accessed 11 June 2019.

National Institute for Health and Care Excellence: Position statement on use of the EQ-5D-5L valuation set for England. https://www.nice.org.uk/about/what-we-do/our-programmes/nice-guidance/technology-appraisal-guidance/eq-5d-5l (2019) Accessed 21 January 2020.

Norwegian Medicines Agency: Guidelines for the submission of documentation for single technology assessment of pharmaceuticals. https://legemiddelverket.no/Documents/English/Public%20funding%20and%20pricing/Documentation%20for%20STA/Guidelines%20151018.pdf (2018) Accessed 11 June 2019.

Pharmaceutical Benefits Advisory Committee, Department of Health and Ageing, Australian Government: Guidelines for preparing a submission to the Pharmaceutical Benefits Advisory Committee (Version 5.0). https://pbac.pbs.gov.au/content/information/files/pbac-guidelines-version-5.pdf (2016) Accessed 11 June 2019.

Pharmaceutical Management Agency (New Zealand): Prescription for pharmacoeconomic analysis. methods for cost-utility analysis (version 2.2). https://www.pharmac.govt.nz/assets/pfpa-2-2.pdf (2015) Accessed 11 June 2019.

Scottish Medicines Consortium: Guidance to Submitting companies for completion of new product assessment form. https://www.scottishmedicines.org.uk/media/4527/20190626-guidance-on-npaf.pdf (2019). Accessed 8 August 2019.

Státní Ústav pro Kontrolu Léčiv (Czech Republic): Cost-effectiveness analysis critical appraisal procedure. https://www.sukl.eu/file/89740_1_2 (2017) Accessed 11 June 2019.

Zorginstituut Nederland: Dutch national guideline for economic evaluations in healthcare. https://english.zorginstituutnederland.nl/publications/reports/2016/06/16/guideline-for-economic-evaluations-in-healthcare (2016) Accessed 11 June 2019.
